# Reliability of a New Swept-Source Optical Coherence Tomography Biometer in Healthy Children, Adults, and Cataract Patients

**DOI:** 10.1155/2020/8946364

**Published:** 2020-05-15

**Authors:** Jinhai Huang, Yuyu Zhao, Giacomo Savini, Guanshun Yu, Jinjin Yu, Zhongxing Chen, Ruixue Tu, Yune Zhao

**Affiliations:** ^1^School of Ophthalmology and Optometry and Eye Hospital, Wenzhou Medical University, Wenzhou, Zhejiang, China; ^2^Key Laboratory of Vision Science, Ministry of Health China, Wenzhou, Zhejiang, China; ^3^G.B. Bietti Foundation IRCCS, Rome, Italy

## Abstract

**Purpose:**

To comprehensively assess the reliability of a new optical biometer (IOLMaster 700), based on swept-source optical coherence tomography (SS-OCT) and comparison with a standard biometer (IOLMaster 500), in healthy children, adults, and cataract patients.

**Methods:**

A total of 301 eyes from 301 consecutive subjects were enrolled prospectively. Two experienced operators measured each eye three times consecutively with the IOLMaster 700. The axial length (AL), keratometry (K), anterior chamber depth (ACD), lens thickness (LT), central corneal thickness (CCT), and white-to-white (WTW) distance were recorded. Intraoperator repeatability and interoperator reproducibility of the IOLMaster 700 were analyzed using the test-retest (TRT), coefficients of variation (CoV), and intraclass correlation coefficients (ICCs). The agreement between the two devices was evaluated using the Bland–Altman method.

**Results:**

The repeatability and reproducibility of the SS-OCT optical biometer were high for all ocular biometry parameters in all groups, except for the WTW in cataract patients (TRT, 0.27–0.44 mm; ICC, 0.86–0.95). The reproducibility of averaged measurements from three consecutive readings (TRT : AL = 0.02 mm, CCT = 5.41 *μ*m, ACD = 0.03 mm, LT = 0.03 mm, Km = 0.17 D, and WTW = 0.22 mm) was higher than the reproducibility of single measurements (TRT : AL = 0.04 mm, CCT = 7.43 *μ*m, ACD = 0.06 mm, LT = 0.05 mm, Km = 0.26 D, and WTW = 0.35 mm) in the three groups. The consistency in the data between the two biometers was high, with narrow 95% LoAs in the three groups.

**Conclusion:**

Repeatability and reproducibility of the new SS-OCT optical biometer were excellent and consistent with that of the standard biometer with respect to healthy children, healthy adults, and cataract patients.

## 1. Introduction

Precise measurements of ocular biometric parameters are crucial for several clinical and research applications in ophthalmology. For example, axial length (AL) is a fundamental parameter for assessing the intraocular lens (IOL) in cataract surgery [1] and refractive lens exchange [2]. A previous study reported that 54% of the error was attributed to AL errors, 8% to corneal power errors, and 38% to errors in the estimation of the postoperative anterior chamber depth (ACD) for predicting the postoperative refractive error [3]. An error in the measurement of AL by only 0.10 mm results in 0.27–0.30 diopters (*D*) of refractive error [4]; this value can vary significantly in myopic and hyperopic eyes [5]. Similarly, deviation in the measurement of keratometry (K) by 1.0 D results in 1.0 *D* of refractive error [4]. Central corneal thickness (CCT) is used to screen candidates for refractive surgery, in order to reduce the risk of postoperative ectasia [6]. CCT readings are also crucial for the detection of contact lens-induced edema and diagnosis of corneal diseases, such as keratoconus and glaucoma [7–9].

For precise measurements, ultrasound biometry has been used and marketed since the 1970s, whereas optical biometry was developed in the late 1990s [10]. A majority of the current optical biometers use time-domain optical coherence tomography (TD-OCT) technology. Recently, a new biometer (IOLMaster 700, Carl Zeiss Meditec AG, Germany) was introduced, based on swept-source OCT (SS-OCT) [11]. The IOLMaster 700 acquires AL measurements using a 1055 nm tunable laser as a light source. The previous version, the IOLMaster 500 (Carl Zeiss Meditec AG), is yet considered as the benchmark among optical biometers to measure the AL of the eye [12].

In contrast to optical biometers based on TD-OCT (including partial coherence interferometry) that use optical A-scans, SS-OCT optical biometers apply optical B-scan technology to determine the biometric data [13]. The optical B-scan technology allows cross-sectional visualization of structures along the visual axis. The IOLMaster 700 can measure the AL, keratometry (K), ACD, lens thickness (LT), CCT, as well as corneal diameter (CD). A few studies have demonstrated that the repeatability and reproducibility of the IOLMaster 700 and the IOLMaster 500 were excellent for measuring the AL, K, and ACD in cataract patients [11, 13].

The subjects of previous studies primarily involved cataract patients. Clinically, healthy children and adults commonly undergo optical biometry, the former in order to assess the evolution of any refractive defect and the latter when they have to consider any refractive surgical procedure. Furthermore, children and elderly do not cooperate easily, rendering variability in the number of measurements and methods for comparing repeatability and reproducibility of previous studies. The present study assessed the intraoperator repeatability and interoperator reproducibility of the measurements provided by the IOLMaster 700 and their agreement with those provided by the IOLMaster 500 in different groups under the same clinical condition. The current study also firstly compared the different groups and methods (average values vs. single values) for comparing the reproducibility.

## 2. Patients and Methods

This prospective study was approved by the Institutional Ethics Committee of the Eye Hospital of Wenzhou Medical University; it followed the tenets of the Declaration of Helsinki. All patients who visited the Eye Hospital of the Wenzhou Medical University at Hangzhou branch provided written informed consent. Only the right eye of each patient was selected for this study. All patients underwent routine preoperative eye examinations including visual acuity, intraocular pressure, slit lamp evaluation, and fundoscopy, along with biometry measurements.

Subjects were assigned to three groups: cataract patients, healthy children in the age group 6–14 years, and healthy adults. The exclusion criteria were as follows: contact lens wear (rigid contact lens within 4 weeks and soft contact lens within 2 weeks), any active ocular pathology, history of ocular surgery and trauma, fundus disease, and systemic diseases with ocular symptoms.

The IOLMaster 700 is a computerized biometry device, which uses SS-OCT technology to acquire the CCT, ACD, LT, and AL from the human eye along the visual axis. The single-step image acquisition utilizes telecentric keratometry with a 950 nm light source such that it is not distance dependent, and the OCT imaging detects the abnormal lens geometries [14]. The device projects the light onto the cornea at 3 zones (1.5, 2.5, and 3.5 mm) with 18 spots; thus, the flattest and steepest K and the mean keratometry (Km) are obtained. The WTW is measured using the LED light source. Furthermore, the device provides a 1.0 mm horizontal OCT scan of the retina to ensure that the measurements on the visual axis are based on the presence of the foveal pit [11]. The standard optical biometer (the IOLMaster 500) measured K from the central cornea in a 2.5 mm zone using six spots of light projected onto the cornea. It uses a 6-point telocentric technique for K readings and an image-based slit lamp system for ACD measurements. Nevertheless, optical A-scans are obtained along the visual axis. However, LT and CCT measurements cannot be measured using the IOLMaster 500 [11]. The WTW was measured using a light emitting diode (LED) light source [15].

The two biometers were placed side-by-side in the same dim room to reduce the examination time, and the measurements were acquired in random sequence without pupil dilation. The right eyes were measured by two independent operators in random sequence using the IOLMaster 700. After forehead and chin placement, the patient was instructed to look at the fixation light in the devices. Next, the subjects were asked to blink immediately before obtaining the measurements to ensure an optically smooth tear film over the cornea. Only high quality and eligible measurements (indicated by a green light in the quality indicator of both biometers) were selected, or else, the measurements were repeated. The two operators performed 3 consecutive measurements to evaluate the intraoperator repeatability. With respect to the interoperator reproducibility of the IOLMaster 700, the average and the first single measurement were calculated by two operators. The first operator utilized the IOLMaster 500 to gain an average value of 5 parameters of the AL and ACD and 3 parameters of K and WTW according to the manufacturer's instructions. The agreement between the two biometers was estimated from the average values of the first operator.

## 3. Statistical Analysis

Statistical analysis was performed using SPSS software (version 21.0, IBM Corporation, USA). Before data analysis, the normality was checked using the Kolmogorov–Smirnov test (*P* > 0.05). The results were presented as the mean standard deviation (SD). Within-subject deviation (Sw), test-retest (TRT) variability, coefficients of variation (CoV), and intraclass correlation coefficients (ICCs) were calculated and analyzed to determine the intraoperator repeatability and interoperator reproducibility of the IOL Master 700. The TRT variability was defined as 2.77 Sw, indicating that the interval within the 95% of the differences between measurements is expected to lie [16]. The CoV was calculated as the ratio of the Sw to the mean (low values indicate superior reliability). A lower CoV was closely related to higher repeatability [17]. The ICCs ranging from 0–1 are commonly classified as follows: <0.75 indicates poor repeatability; 0.75 to <0.90 indicates moderate repeatability; ≥0.90 indicates high repeatability [17].

To assess the agreement between the two devices, a paired *t*-test was performed (*P* < 0.05 was statistically significant) and Bland–Altman plots were calculated [18]. This method involved plotting the difference between the two methods against their mean, thereby allowing an assessment of the systematic difference between measurements (i.e., fixed bias). The mean difference is the estimated bias, and the standard deviation (SD) of the differences measures the random fluctuations of this mean. The 95% limits of agreement (LoA) were defined as means ± 1.96 SD of the differences between the two measurement techniques. A *P* value <0.05 was considered as statistically significant.

## 4. Results

109 eyes from 109 cataract patients (38 men) with a mean age of 71.07 ± 10.44 (range, 35–92) years, 100 eyes from healthy children (39 boys) with a mean age of 10.37 ± 1.81 (range, 7–14) years, and 92 eyes from healthy adults (27 men) with a mean age of 28.90 ± 6.40 (range, 20–48) years were included in the study.

The AL, Km, ACD, LT, CCT, and WTW measurements by the IOLMaster 700 revealed high intraobserver repeatability for each operator in the three groups (Tables 1–3). The CoVs were <0.73%, except WTW (<1.25%). The ICCs of all measured parameters were >0.97, except for WTW. The measurement of the AL provided maximal repeatability as the TRT variability was <0.04 mm and the ICC was 1.0. The lens thickness ranked from thick to thin in cataract patients (4.61 ± 0.37 mm), healthy adults (3.75 ± 0.27 mm), and healthy children (3.38 ± 0.15 mm). With the first operator, the TRT variability and the CoVs for LT measurements were the lowest in cataract patients (0.02 mm, 0.13%), followed by healthy adult subjects (0.04 mm, 0.41%), whereas the worst results were obtained in children (0.07 mm, 0.72%). The second operator showed a similar pattern.

Tables 4–6 show the interobserver reproducibility of all parameters obtained by averaging the three consecutive readings from each operator and the single IOLMaster 700 reading from each operator in the three groups. Overall, the TRT variability of single measurements was 100% higher than the TRT variability of the averaged measurements for AL, 37.3% for CCT, 100% for ACD, 66.7% for LT, 52.9% for Km, and 59% for WTW. A similar trend was noted in cataract patients, healthy adults, and children. Furthermore, the reproducibility of single measurements, as defined by Sw, TRT variability, and CoV, was poor than the reproducibility of the averaged measurements of all parameters. The TRT values of reproducibility were excellent for their low variability, <0.02 mm for AL, 5.47 *μ*m for CCT, 0.04 mm for ACD, 0.05 mm for LT, 0.19 *D* for Km, and 0.27 mm for WTW. The CoVs were always <0.82%, and the ICCs were approximately 1 in each case.

**Figure 1:**
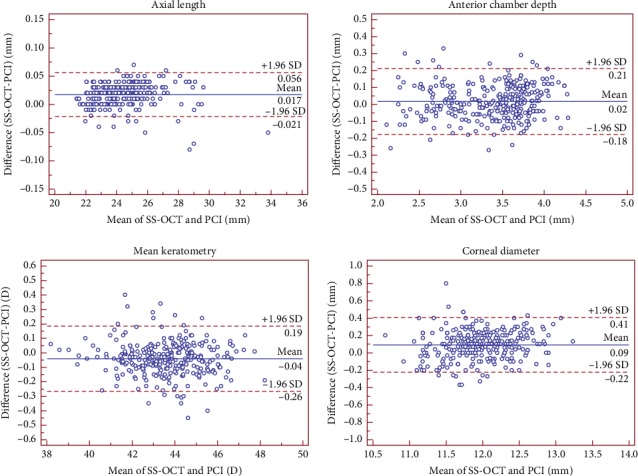
Bland–Altman plot of the axial length, anterior chamber depth, mean keratometry and corneal diameter measurements using the IOLMaster 700 swept-source optical coherence tomography against IOLMaster partial coherence interferometry. The solid line represents the mean difference. The dotted lines on the side represent the upper and lower 95% LoA.

**Figure 2:**
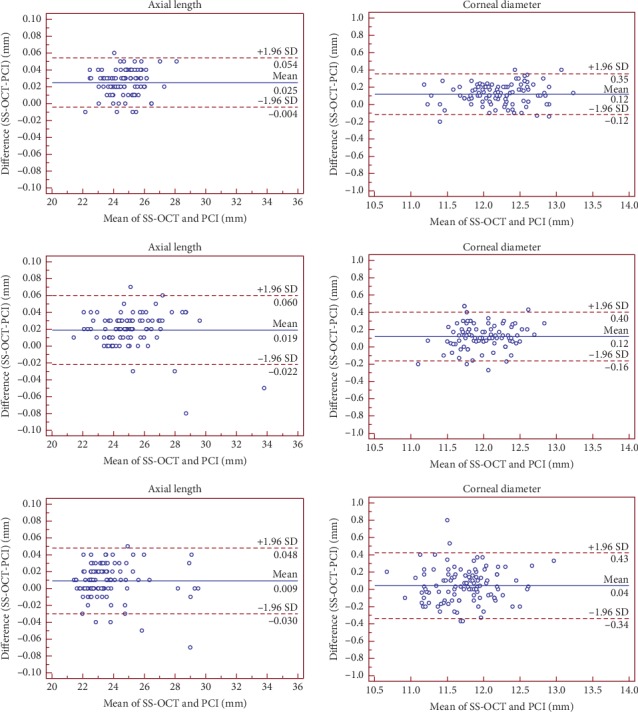
Bland–Altman plot of the axial length measurements and the corneal diameter measurements using IOLMaster 700 swept-source optical coherence tomography against IOLMaster partial coherence interferometry in children (in the first row), normal subjects (in the second row), and cataract patients (in the third row). The solid line represents the mean difference. The dotted lines on the side represent the upper and lower 95% LoA.

The mean differences were approximately zero. The AL and WTW values were slightly higher with the IOLMaster 700 than with the IOLMaster 500, whereas Km values were slightly lower. Bland–Altman plots (Figures 1 and 2) showed relatively narrow ranges (AL: −0.03–0.06 mm, ACD: −0.21–0.24 mm, and Km: −0.30–0.26 D), which implied excellent agreement between the two biometers. Bland–Altman plots of WTW in cataract patients showed relatively low agreement.

## 5. Discussion

With cataract surgery as one of the most commonly performed procedures worldwide, assessment of the devices used in this field is critical for advances in the field and improving the outcomes of patients [19, 20]. Furthermore, with increasing expectation with laser in situ keratomile uses (LASIK) in adult patient eyes with myopic astigmatism, the final refractive outcome is of utmost importance [21, 22]. In addition, increased AL and corneal curvature are the significant reasons for myopic progression in schoolchildren [23, 24]. Thus, biometers are key for providing an accurate geometric measurement of the eye in cataract patients, myopic patients, and schoolchildren.

In the present study, for the first time, we investigated the repeatability and reproducibility of IOLMaster 700 and the agreement with IOLMaster 500 with respect to the measurements in cataract patients, healthy children, and adults in a large sample under similar clinical conditions. Furthermore, we compared the different methods (average values vs. single values) for assessing reproducibility. The current comprehensive prospective study confirmed the excellent repeatability and reproducibility as well as the agreement between the 2 biometers regarding all ocular biometry parameters in the three groups.

Anatomical parameters, especially AL is fundamental to the accurate determination of the most suitable IOL power at the time of cataract surgery. In addition, increased AL is the most significant reason for myopic progression in myopic adults and children. AL showed the highest repeatability and reproducibility (TRT, 0.01–0.04 mm; ICC, 1.0) in the three groups that were consistent with the results of the studies by Srivannaboon et al. [11], Ferrer-Blasco et al. [25], and Sel et al [26]. Given that in a normal eye, a 0.10 mm error in AL is equivalent to an error of about 0.27 D in the spectacle plane [27], and the errors in the refractive prediction due to AL variability are negligible. The agreement values of the IOLMaster 500 were high with the mean difference <0.02 mm and 95% LoAs as −0.03 to 0.06 mm in the three groups.

The high ICC (0.99–1.0) and low TRT (0.02–0.06 mm) of the SS-OCT demonstrate the repeatability and reproducibility of the instrument for the measurement of ACD. Several formulae, such as those by Haigis, Olsen, and Barrett, rely on this parameter to estimate the IOL position [28, 29]. The ACD measurement is critical for the early diagnosis of angle-closure glaucoma and the estimation of the course of disease [30].

Between the two devices, only the keratometry measurement was derived using the same technology and the distance-independent telecentric keratometer. The IOLMaster 700 projects the light onto the cornea at 3 zones (1.5, 2.5, and 3.5 mm) with 18 spots. Using low TRT and low CoV, a precise measurement of the corneal power was obtained, which is better than the OA-2000 [31, 32]. Compared to the IOLMaster 500, which measures the central corneal curvature at 2.5 mm, Bland–Altman plots showed that the agreement between the two biometers was high in the three groups (−0.30 to 0.26 D). In clinical practice, even small differences in K measurements require constant optimization for any IOL model and formula [33]. In addition, Km is a vital evaluation factor for keratoconus [34, 35], IOL power calculation, and refractive surgery, such as LASIK.

Unlike the PCI, the IOLMaster 700 could measure CCT and LT, which were repeatable and reproducible in the three groups in this study. The repeatability and reproducibility of CCT values were similar to that previously reported with another device using SS-OCT, the OA-2000 with ICC (0.982–0.987) and TRT (10.61–12.28 *μ*m) for CCT measurements in normal subjects [31]. The high ICC at 0.994 and low TRT of the IOLMaster 700 demonstrated excellent repeatability and reproducibility of the instrument for the measurement of CCT. In the case of LT, we minimized the role of accommodation using the same interior target for a single subject. The IOLMaster 700 enabled accurate calculation of LT for IOL power [36, 37], and lens densitometry was utilized to assess the severity of cataract [38].

In addition, we found high repeatability and reproducibility of measurements for all ocular biometrical parameters with low TRTs and high ICCs (>0.973), except for the WTW in cataract patients (TRT <0.41 mm; ICC >0.893). The relatively low repeatability and reproducibility of WTW in cataract patients may be related to the occurrence of corneal arcus. A transparent area (width <1 mm) is present between this ring and the cornea. The boundaries were distinct, closer to the cornea, and dimmer in the center [39]. The corneal arcus is a risk factor for cataract phacoemulsification coupled with IOL implantation.

The data between the IOLMaster 700 and IOLMaster 500 were high as shown by the narrow 95% LoAs of AL, ACD, and Km in the three groups. Although the precision of WTW measurements by the SS-OCT was high, the agreement with the PCI in cataract patients was low with respect to the other parameters. A relatively poor agreement for WTW measurements has been reported when comparing the PCI with other devices such as the OA-2000 [31, 40] and AL-scan [41]; similar findings were reported by Srivannaboon et al [11]. Furthermore, with respect to AL in the three groups, the mean difference was small between the two devices, and hence, these could be clinically interchangeable.

Nevertheless, the present study has some limitations. First, we only assessed the reliability of measurements with IOLMaster 700 in cataract patients. Therefore, we could not assess the rate of failure in measuring AL due to opacities. Moreover, we only recruited children >6 years old, and young children did not cooperate with these measurements easily. These limitations will be addressed in a future study.

## 6. Conclusions

In conclusion, the new SS-OCT-based optical biometer (the IOLMaster 700) provides repeatable and reproducible measurements of AL, CCT, LT, ACD, K, and WTW, which are consistent with those obtained with the IOLMaster 500. Thus, it is considered a reliable option to obtain the accurate geometric measurement. In addition, we recommend that clinicians use the average values determined from each operator's 3 consecutive readings to assess the interobserver reproducibility.

## Figures and Tables

**Table 1:** Intraobserver repeatability outcomes for biometric measurements obtained using IOLMaster 700 swept-source optical coherence tomography in children.

Parameter	Observer	Mean ± SD	*S*_w_	TRT	CoV (%)	ICC (95% CI)
AL (mm)	1st	24.7 ± 1.07	0.01	0.03	0.04	1.000 (1.000 to 1.000)
	2nd	24.7 ± 1.07	0.01	0.02	0.03	1.000 (1.000 to 1.000)
CCT (*μ*m)	1st	552.27 ± 33.6	2.80	7.76	0.51	0.993 (0.990 to 0.995)
	2nd	551.78 ± 33.2	2.86	7.93	0.52	0.993 (0.989 to 0.995)
ACD (mm)	1st	3.76 ± 0.24	0.02	0.05	0.48	0.994 (0.992 to 0.996)
	2nd	3.76 ± 0.23	0.01	0.04	0.40	0.996 (0.994 to 0.997)
LT (mm)	1st	3.38 ± 0.15	0.02	0.07	0.72	0.973 (0.962 to 0.981)
	2nd	3.38 ± 0.15	0.02	0.06	0.61	0.981 (0.974 to 0.987)
Km (D)	1st	42.73 ± 1.55	0.09	0.26	0.22	0.996 (0.995 to 0.997)
	2nd	42.73 ± 1.55	0.11	0.30	0.25	0.995 (0.993 to 0.997)
WTW (mm)	1st	12.28 ± 0.43	0.09	0.26	0.75	0.955 (0.938 to 0.968)
	2nd	12.27 ± 0.42	0.09	0.26	0.76	0.952 (0.934 to 0.966)

AL = axial length, CCT = central corneal thickness, ACD = anterior chamber depth, LT = lens thickness, *K* = keratometry, WTW = white to white, SD = standard deviation, *S*_w_ = within-subject standard deviation, TRT = test-retest repeatability (2.77 *S*_w_), CoV = within-subject coefficient of variation, and ICC = intraclass correlation coefficient.

**Table 2:** Intraobserver repeatability outcomes for biometric measurements obtained using IOLMaster 700 swept-source optical coherence tomography in normal subjects.

Parameter	Observer	Mean ± SD	*S*_w_	TRT	CoV (%)	ICC (95% CI)
AL (mm)	1st	25.1 ± 1.85	0.01	0.02	0.02	1.000 (1.000 to 1.000)
	2nd	25.1 ± 1.85	0.01	0.02	0.03	1.000 (1.000 to 1.000)
CCT (*μ*m)	1st	542.84 ± 31.96	2.21	6.11	0.41	0.995 (0.993 to 0.997)
	2nd	542.54 ± 32.13	2.20	6.10	0.41	0.995 (0.993 to 0.997)
ACD (mm)	1st	3.56 ± 0.27	0.01	0.04	0.40	0.997 (0.996 to 0.998)
	2nd	3.56 ± 0.28	0.02	0.04	0.43	0.997 (0.996 to 0.998)
LT (mm)	1st	3.75 ± 0.27	0.02	0.04	0.41	0.997 (0.996 to 0.998)
	2nd	3.75 ± 0.27	0.02	0.05	0.45	0.996 (0.995 to 0.997)
Km (D)	1st	43.72 ± 1.36	0.10	0.28	0.23	0.994 (0.992 to 0.996)
	2nd	43.73 ± 1.35	0.09	0.25	0.21	0.996 (0.994 to 0.997)
WTW (mm)	1st	12.04 ± 0.35	0.09	0.24	0.72	0.940 (0.917 to 0.958)
	2nd	12.04 ± 0.37	0.09	0.25	0.74	0.943 (0.920 to 0.960)

AL = axial length, CCT = central corneal thickness, ACD = anterior chamber depth, LT = lens thickness, *K* = keratometry, WTW = white to white, SD = standard deviation, *S*_w_ = within-subject standard deviation, TRT = test-retest repeatability (2.77 *S*_w_), CoV = within-subject coefficient of variation, and ICC = intraclass correlation coefficient.

**Table 3:** Intraobserver repeatability outcomes for biometric measurements obtained using IOLMaster 700 swept-source optical coherence tomography in cataract patients.

Parameter	Observer	Mean ± SD	*S*_w_	TRT	CoV (%)	ICC (95% CI)
AL (mm)	1st	23.6 ± 1.73	0.02	0.04	0.07	1.000 (1.000 to 1.000)
	2nd	23.6 ± 1.73	0.01	0.02	0.04	1.000 (1.000 to 1.000)
CCT (*μ*m)	1st	534.09 ± 32.64	2.48	6.88	0.46	0.994 (0.992 to 0.996)
	2nd	533.69 ± 32.44	2.61	7.24	0.49	0.994 (0.991 to 0.995)
ACD (mm)	1st	2.87 ± 0.36	0.01	0.02	0.25	1.000 (0.999 to 1.000)
	2nd	2.88 ± 0.36	0.02	0.06	0.73	0.997 (0.995 to 0.998)
LT (mm)	1st	4.61 ± 0.37	0.01	0.02	0.13	1.000 (1.000 to 1.000)
	2nd	4.61 ± 0.37	0.01	0.02	0.16	1.000 (0.999 to 1.000)
Km (D)	1st	44.29 ± 1.57	0.09	0.26	0.21	0.996 (0.995 to 0.997)
	2nd	44.28 ± 1.57	0.10	0.28	0.23	0.996 (0.994 to 0.997)
WTW (mm)	1st	11.76 ± 0.41	0.12	0.33	1.01	0.922 (0.895 to 0.944)
	2nd	11.75 ± 0.43	0.15	0.41	1.25	0.893 (0.856 to 0.922)

AL = axial length, CCT = central corneal thickness, ACD = anterior chamber depth, LT = lens thickness, *K* = keratometry, WTW = white to white, SD = standard deviation, *S*_w_ = within-subject standard deviation, TRT = test-retest repeatability (2.77 *S*_w_), CoV = within-subject coefficient of variation, and ICC = intraclass correlation coefficient.

**Table 4:** Interobserver reproducibility outcomes for biometric measurements obtained using an IOLMaster 700 swept-source optical coherence tomography-based biometer in children.

Parameter	Methods	*S*_w_	TRT	CoV (%)	ICC
AL (mm)	Average	0.005	0.01	0.02	1.000 (1.000 to 1.000)
	Single	0.01	0.02	0.03	1.000 (1.000 to 1.000)
CCT (*μ*m)	Average	1.96	5.42	0.35	0.997 (0.995 to 0.998)
	Single	2.59	7.18	0.47	0.994 (0.991 to 0.996)
ACD (mm)	Average	0.01	0.04	0.34	0.997 (0.996 to 0.998)
	Single	0.02	0.05	0.52	0.993 (0.989 to 0.995)
LT (mm)	Average	0.02	0.05	0.52	0.986 (0.979 to 0.991)
	Single	0.03	0.07	0.75	0.969 (0.954 to 0.979)
Km (D)	Average	0.05	0.14	0.12	0.999 (0.998 to 0.999)
	Single	0.09	0.25	0.21	0.997 (0.995 to 0.998)
CD (mm)	Average	0.07	0.18	0.54	0.976 (0.965 to 0.984)
	Single	0.10	0.29	0.85	0.943 (0.917 to 0.962)

AL = axial length, CCT = central corneal thickness, ACD = anterior chamber depth, LT = lens thickness, Km = mean keratometry, CD = corneal diameter, SD = standard deviation, *S*_w_ = within-subject standard deviation, TRT = test-retest repeatability (2.77 *S*_w_), CoV = within-subject coefficient of variation, and ICC = intraclass correlation coefficient.

**Table 5:** Interobserver reproducibility outcomes for biometric measurements obtained using an IOLMaster 700 swept-source optical coherence tomography-based biometer in normal subjects.

Parameter	Methods	*S*_w_	TRT	CoV (%)	ICC
AL (mm)	Average	0.01	0.01	0.02	1.000 (1.000 to 1.000)
	Single	0.01	0.02	0.03	1.000 (1.000 to 1.000)
CCT (*μ*m)	Average	1.97	5.47	0.36	0.996 (0.994 to 0.997)
	Single	2.63	7.30	0.49	0.993 (0.990 to 0.995)
ACD (mm)	Average	0.01	0.03	0.28	0.999 (0.998 to 0.999)
	Single	0.02	0.04	0.43	0.997 (0.995 to 0.998)
LT (mm)	Average	0.01	0.03	0.32	0.998 (0.997 to 0.999)
	Single	0.02	0.05	0.47	0.996 (0.994 to 0.997)
Km (D)	Average	0.07	0.19	0.15	0.998 (0.996 to 0.998)
	Single	0.10	0.27	0.23	0.995 (0.992 to 0.996)
CD (mm)	Average	0.06	0.18	0.53	0.968 (0.952 to 0.979)
	Single	0.10	0.26	0.79	0.929 (0.895 to 0.953)

AL = axial length, CCT = central corneal thickness, ACD = anterior chamber depth, LT = lens thickness, Km = mean keratometry, CD = corneal diameter, SD = standard deviation, *S*_w_ = within-subject standard deviation, TRT = test-retest repeatability (2.77 *S*_w_), CoV = within-subject coefficient of variation, and ICC = intraclass correlation coefficient.

**Table 6:** Interobserver reproducibility outcomes for biometric measurements obtained using an IOLMaster 700 swept-source optical coherence tomography-based biometer in cataract patients.

Parameter	Methods	*S*_w_	TRT	CoV (%)	ICC
AL (mm)	Average	0.01	0.02	0.03	1.000 (1.000 to 1.000)
	Single	0.02	0.05	0.08	1.000 (1.000 to 1.000)
CCT (*μ*m)	Average	1.93	5.36	0.36	0.996 (0.995 to 0.998)
	Single	2.80	7.77	0.53	0.993 (0.989 to 0.995)
ACD (mm)	Average	0.01	0.03	0.34	0.999 (0.999 to 0.999)
	Single	0.03	0.07	0.92	0.995 (0.992 to 0.996)
LT (mm)	Average	0.00	0.01	0.09	1.000 (1.000 to 1.000)
	Single	0.01	0.02	0.16	1.000 (0.999 to 1.000)
Km (D)	Average	0.07	0.18	0.15	0.998 (0.997 to 0.999)
	Single	0.09	0.26	0.21	0.996 (0.995 to 0.998)
CD (mm)	Average	0.10	0.27	0.82	0.948 (0.925 to 0.964)
	Single	0.16	0.44	1.36	0.863 (0.805 to 0.904)

AL = axial length, CCT = central corneal thickness, ACD = anterior chamber depth, LT = lens thickness, Km = mean keratometry, CD = corneal diameter, SD = standard deviation, *S*_w_ = within-subject standard deviation, TRT = test-retest repeatability (2.77 *S*_w_), CoV = within-subject coefficient of variation, and ICC = intraclass correlation coefficient.

## Data Availability

Data pertaining to this manuscript will be made available upon request.
